# NRP/Optineurin Cooperates with TAX1BP1 to Potentiate the Activation of NF-κB by Human T-Lymphotropic Virus Type 1 Tax Protein

**DOI:** 10.1371/journal.ppat.1000521

**Published:** 2009-07-17

**Authors:** Chloé Journo, Josina Filipe, Frédégonde About, Sébastien A. Chevalier, Philippe V. Afonso, John N. Brady, David Flynn, Frédéric Tangy, Alain Israël, Pierre-Olivier Vidalain, Renaud Mahieux, Robert Weil

**Affiliations:** 1 Unité d'Epidémiologie et Physiopathologie des Virus Oncogènes, CNRS URA 3015, Institut Pasteur, Paris, France; 2 Oncogenèse Rétrovirale, INSERM U758, Lyon, France; 3 Ecole Normale Supérieure, Lyon, France; 4 IFR 128 BioSciences Lyon-Gerland, Lyon, France; 5 Unité de Signalisation Moléculaire et Activation Cellulaire, CNRS URA 2582, Institut Pasteur, Paris, France; 6 Laboratory of Cellular Oncology, NIH/NCI, Bethesda, Maryland, United States of America; 7 Laboratoire de Génomique Virale et Vaccination, Institut Pasteur, Paris, France; University of Pennsylvania School of Medicine, United States of America

## Abstract

Nuclear factor (NF)-κB is a major survival pathway engaged by the Human T-Lymphotropic Virus type 1 (HTLV-1) Tax protein. Tax1 activation of NF-κB occurs predominantly in the cytoplasm, where Tax1 binds NF-κB Essential Modulator (NEMO/IKKγ) and triggers the activation of IκB kinases. Several independent studies have shown that Tax1-mediated NF-κB activation is dependent on Tax1 ubiquitination. Here, we identify by co-immunoprecipitation assays NEMO-Related Protein (NRP/Optineurin) as a binding partner for Tax1 in HTLV-1 infected and Tax1/NRP co-expressing cells. Immunofluorescence studies reveal that Tax1, NRP and NEMO colocalize in Golgi-associated structures. The interaction between Tax1 and NRP requires the ubiquitin-binding activity of NRP and the ubiquitination sites of Tax1. In addition, we observe that NRP increases the ubiquitination of Tax1 along with Tax1-dependent NF-κB signaling. Surprisingly, we find that in addition to Tax1, NRP interacts cooperatively with the Tax1 binding protein TAX1BP1, and that NRP and TAX1BP1 cooperate to modulate Tax1 ubiquitination and NF-κB activation. Our data strongly suggest for the first time that NRP is a critical adaptor that regulates the assembly of TAX1BP1 and post-translationally modified forms of Tax1, leading to sustained NF-κB activation.

## Author Summary

Oncogenic viruses (i.e., viruses that can induce cancer) have usually been found to deregulate several cellular signaling pathways controlling cell survival and proliferation. Among those, the NF-κB pathway is particularly important. In this study, we focus on the Human T-Lymphotropic Virus type 1 (HTLV-1), which infects immune T cells, and is associated with the development of a severe hematological disease, termed adult T cell leukemia. The viral Tax oncoprotein is known to activate the NF-κB pathway, but the precise mechanism is still under investigation. In cells, proteins can undergo modifications that can modulate their function. In the case of Tax, a modified form of the protein (ubiquitinated Tax) is able to activate the NF-κB pathway. Our aim was to identify cellular proteins that participate in the modification of Tax, and in turn in the regulation of its function. We show for the first time that the cellular protein NRP/Optineurin interacts with Tax and increases its ubiquitination, thus leading to an enhanced NF-κB activation. We further demonstrate that TAX1BP1, another cellular protein that had been previously identified as a partner of Tax, also participates in this regulation. Thus, this study uncovers new actors of the virally induced cell signaling.

## Introduction

Human T-Lymphotropic Virus type 1 (HTLV-1) is the etiological agent of Adult T cell Leukemia/Lymphoma (ATL) and of HTLV-Associated Myelopathy/Tropical Spastic Paraparesis (HAM/TSP) [1]–[3]. HTLV-1 contains a unique pX region in the 3′ portion of its genome, which encodes regulatory and accessory proteins that are involved in viral replication and cell proliferation. Among them, Tax1 plays a critical role by triggering cell immortalization through various mechanisms [4], including activation of signaling pathways such as NF-κB [5].

The NF-κB family of transcription factors plays an important role in the regulation of cellular activation, proliferation, and survival. A large number of stimuli including bacterial lipopolysaccharide (LPS), tumor necrosis factor (TNF)-α, interleukin (IL)-1 and antigens can activate NF-κB. NF-κB activity is tightly regulated by inhibitory IκB proteins. Upon stimulation, signals are transduced that lead to the degradation of IκB, allowing NF-κB to translocate into the nucleus and to activate its target genes. IκB degradation by the 26S proteasome is triggered by its phosphorylation by a multisubunit IκB kinase (IKK) complex that contains two homologous catalytic subunits (IKKα and IKKβ) and a regulatory subunit, NF-κB Essential Modulator (NEMO/IKKγ). An important mechanism in the NF-κB pathway is the interaction between NEMO and K63-linked polyubiquitin chains. In the case of TNF-α stimulation, the attachment of the polyubiquitin chains to RIP1 serves to bring the NEMO/IKK complex to the TNF-α receptor and is required for NF-κB activation [6]. Other studies have shown that TCR and IL-1 stimulations induce the attachment of K63-linked polyubiquitin chains to Bcl10 and IRAK1 respectively, which are required for binding to NEMO and subsequent activation of NF-κB [7],[8]. One of the main mechanisms restricting this process is the NF-κB-mediated induction of deubiquitinases such as A20 and CYLD [9],[10].

NF-κB activation plays a critical role in HTLV-1-mediated oncogenesis. This process occurs predominantly in the cytoplasm where HTLV-1 Tax1 binds NEMO and triggers the activation of IKKα and IKKβ [11]–[13]. Tax1 can also stimulate the alternative pathway of NF-κB activation through the IKKα-dependent processing of NF-κB p100 precursor protein [14]. Independent studies have shown that Tax1 ubiquitination is dependent on the E2 ubiquitin-conjugating enzyme Ubc13 and is critical for Tax1 binding to NEMO and the subsequent NF-κB activation [15]–[18]. In addition, Tax1 binding protein TAX1BP1 [19],[20] is involved in the recruitment of A20 deubiquitinase and the negative control of TNF-α-, IL-1- and LPS-mediated NF-κB activation [17], suggesting that Tax1-dependent activation of NF-κB could also be more complex than originally thought. Because cellular factors other than NEMO and Ubc13 could contribute to the activation of NF-κB by Tax1, we searched for novel interactors of Tax1 and Tax2, the equivalent of Tax1 for HTLV-2. Here we report the identification of NEMO-Related Protein (NRP) as a novel Tax interactor. NRP is ubiquitously expressed and exhibits strong homologies to NEMO (53% sequence similarity), but its function is still unknown [21]. Mutations in its sequence have been associated with primary open-angle glaucoma (POAG), and for this reason, NRP was also named Optineurin for “optic neuropathy inducing” protein [22].

We show that both NRP and TAX1BP1 form a functional complex with Tax1, and we demonstrate that a synergistic interaction between TAX1BP1 and NRP contributes to Tax1-mediated NF-κB activation.

## Results

### Tax2 interacts with NRP in a yeast two-hybrid screen

To identify novel binding partners of Tax proteins, we used a standard yeast two-hybrid screening procedure. Full-length Tax1 or Tax2 proteins were fused to Gal4 DNA binding domain (Gal4-BD) and used as baits to screen a library of human spleen cDNA fused to Gal4 transactivation domain (Gal4-AD). Screens were performed by mating to reach a five-time coverage of the cDNA library complexity, and yielded 59 positive yeast colonies with Tax1 and 36 with Tax2. Using Tax1 as bait, we retrieved three previously known interactors of Tax1 [TAX1BP1 (2 colonies), TAX1BP3 (37 colonies) and SRF (3 colonies)], which demonstrates the specificity of the screen. NEMO was only found using Tax2 as bait (2 colonies). This corresponds to a known limitation of the two-hybrid system since numerous pairs of interacting proteins fail to rebuild a functional transcription factor in yeast when fused to Gal4-DB and Gal4-AD. From the screen performed with Tax2, we essentially selected yeast colonies expressing a new interactor of Tax: NEMO-Related Protein NRP (23 colonies). Although various lengths of the NRP protein were encoded, the smallest NRP fragment shown to interact with Tax2 was spanning amino acid 411 to the C-terminus end of the protein. This led us to test the ability of Tax1 to interact with NRP using a different binding assay.

### Both Tax1 and Tax2 proteins co-precipitate with NRP

**Figure 1:**
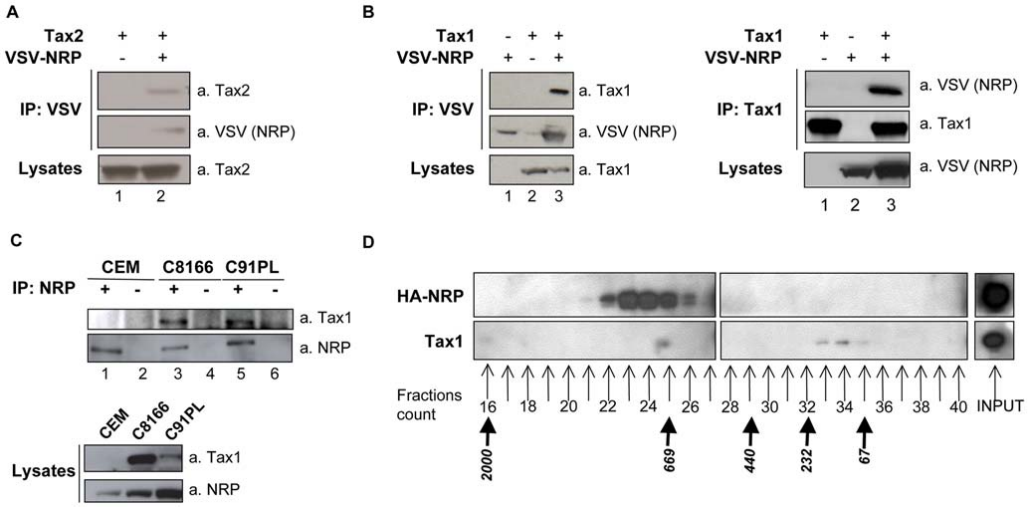
Co-immunoprecipitation and co-fractionation of Tax and NRP. (A) 293T cells were transfected with Tax2 and VSV-NRP as indicated. Total lysates were immunoprecipitated with an anti-VSV antibody and western blot analysis was performed using anti-Tax2 and anti-VSV antibodies. (B) 293T cells were transfected with Tax1 and VSV-NRP as indicated. Total lysates were immunoprecipitated with an anti-VSV or anti-Tax1 antibody and western blot analysis was performed using anti-Tax1 and anti-VSV antibodies. (C) Total lysates from CEM, C8166 and C91PL cell lines were immunoprecipitated with an anti-NRP antibody (+) or an irrelevant anti-His antibody (−) and western blot analysis was performed using anti-NRP and anti-Tax1 antibodies. (D) HeLa cells were transfected with Tax1 and HA-NRP, and cell extracts were analyzed by gel filtration chromatography. Fractions 16 to 40 as well as total extracts (input) were then analyzed by western blotting using antibodies directed against HA (upper panel) or Tax1 (lower panel). Precalibration of the column is indicated beneath the fractions count (kDa).

In order to confirm Tax/NRP interaction, Tax1 and Tax2 were co-expressed in 293T cells with VSV-tagged NRP and co-immunoprecipitations were performed. Both Tax2 (Figure 1A, lane 2) and Tax1 (Figure 1B, left panel, lane 3) were detected in VSV-NRP immunoprecipitates.

The reverse experiment (i.e. immunoprecipitation of Tax1 followed by immunoblotting for VSV-NRP) confirmed the interaction between the two proteins (Figure 1B, right panel, lane 3). We then aimed to demonstrate the interaction between Tax1 and endogenous NRP in two HTLV1-infected cell lines, C8166 and C91PL. The uninfected cell line CEM was used here as a negative control. As expected, Tax1 could be specifically recovered from NRP immunoprecipitates in both HTLV-1-infected cell lines (Figure 1C, compare lanes 3 and 5 with lane 1). The specificity of these interactions was controlled using an irrelevant antibody for the immunoprecipitation (Figure 1C, lanes 2, 4 and 6). Hence, NRP interacts with Tax1 both in transfected and in infected cells.

In order to confirm the interaction with an alternative biochemical method, we determined whether Tax1 and NRP could be found in the same fractions after gel filtration (Figure 1D). Experiments using glycerol gradients were also performed (Figure S1). Tax1 and HA-tagged NRP were co-expressed in HeLa cells. After gel filtration, extracts were analyzed by western blot. As shown in Figure 1D, Tax1 was recovered from fractions 33 to 35 (Figure 1D, lower panel), corresponding to low molecular mass fractions. This subset of Tax1 molecules essentially represents free molecules. Tax1 was also recovered from fraction 25, suggesting that a subset of Tax1 molecules was present in high molecular mass complexes. HA-NRP was recovered from fractions 22 to 26 (Figure 1D, upper panel), showing that a majority of HA-NRP molecules were found in high molecular mass complexes. Co-fractionation of Tax1 and HA-NRP in fraction 25 indicated that both proteins could be found in the same complexes.

These results obtained with distinct biochemical methods support the interaction between Tax1 and NRP.

### Tax1 and NRP have the same subcellular distribution

**Figure 2:**
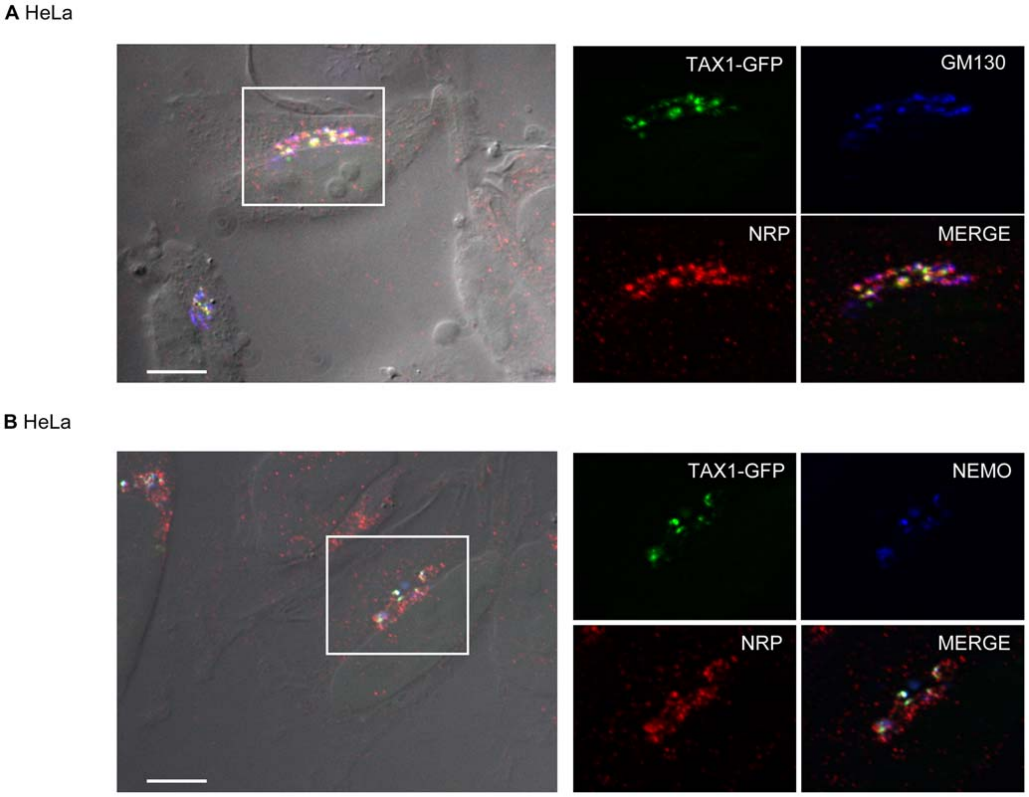
Colocalization of Tax1, NRP and NEMO in Golgi-associated structures in HeLa cells. HeLa cells were transfected with Tax1-GFP. Cells were stained with an anti-NRP antibody (red) and either (A) an anti-GM130 or (B) an anti-NEMO antibody (blue). Cells were observed as described in the Materials and Methods section. Differential interference contrast is shown on the left merge image. Scale bar = 10 µm.

Tax1 has been described as having distinct localizations depending on its post-translational modification status [15],[16]. Ubiquitinated Tax1 was reported to localize in the cytoplasm, more specifically in Golgi/centrosome-associated structures [18], where it interacts with NEMO and induces NF-κB activation. SUMOylated Tax1, however, was found in the nucleus [15],[16]. NRP, on the other hand, is predominantly localized at the Golgi apparatus [21]. Since Tax1 was shown to promote the relocalization of the NEMO/IKK complex to the Golgi apparatus [23], we suspected that NRP might also interact with Tax1 at the Golgi apparatus. We performed a series of immunofluorescence stainings in HeLa cells expressing a C-terminal GFP-tagged Tax1 plasmid, which was previously demonstrated to have a subcellular localization and an ability to activate NF-κB similar to those of untagged Tax1 ([24] and data not shown). To evaluate whether NRP colocalizes with Tax1-GFP and NEMO at the Golgi apparatus, we performed a double staining for NRP and either NEMO or GM130 in order to visualize the Golgi apparatus (Figure 2). As previously described, Tax1-GFP showed a discrete granular appearance in the cytoplasm, which was more intense at the Golgi apparatus as shown by colocalization with GM130 staining (Figure 2A, green and blue). Staining for NEMO indicated that expression of Tax1-GFP induced the recruitment of this protein at the Golgi apparatus where both proteins colocalize (Figure 2B, green and blue). As expected, NRP was predominantly localized at the Golgi apparatus, and its localization was not affected by Tax1-GFP expression (Figure 2A and B, red). Interestingly, we also observed colocalization between Tax1-GFP and NRP in these Golgi-associated structures (Figure 2A and B).

Because HTLV-1 infects mainly T cells *in vivo*, we performed similar immunofluorescence microscopy studies using Jurkat T cells (Figure S2). Consistent with the results obtained in HeLa cells, a subset of Tax1-positive cells harbored a cytoplasmic staining for Tax1-GFP, which colocalized with NRP (Figure S2A and B, red) and NEMO (Figure S2B, blue) in perinuclear structures that were associated with the Golgi apparatus (Figure S2A, blue). As in HeLa cells, we observed that Tax1-GFP expression had no effect on NRP localization (Figure S2, compare A and C).

These results are consistent with the observed interaction between Tax1 and NRP *in vivo*, and suggest that these interactions occur at the Golgi apparatus where NEMO is recruited. Because it has been reported that Tax1 interacts with NEMO at the Golgi apparatus in an ubiquitin-dependent manner, we hypothesized that a similar mechanism could be involved in the Tax1/NRP interaction.

### NRP ubiquitin-binding domain (UBD) mediates binding to Tax1

**Figure 3:**
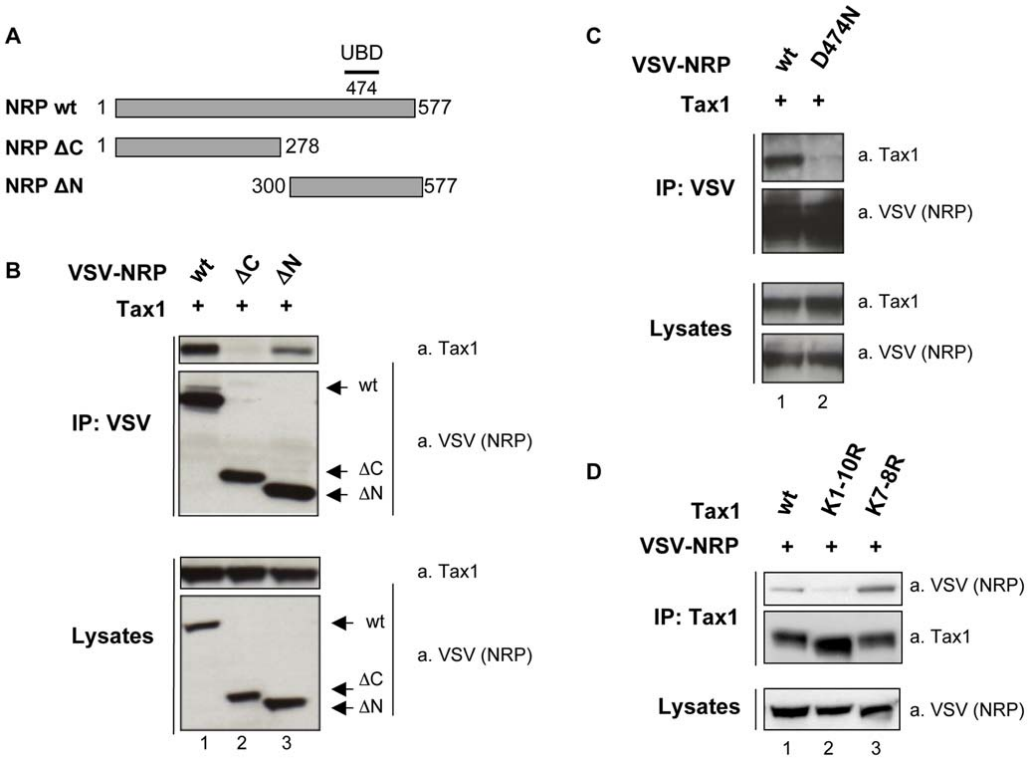
Involvement of NRP UBD and Tax1 ubiquitination in NRP/Tax1 interaction. (A) Schematic representation of NRP constructs used in the experiment. NRP ΔC construct is deleted from amino acid 279 to the C-terminus of the protein, whereas NRP ΔN is deleted from amino acid 1 to 299. Substitution at position 474 is known to abrogate the function of the ubiquitin-binding domain (UBD). 293T cells were transfected with (B) wild-type Tax1 and either wild-type VSV-NRP (wt), VSV-NRP ΔC, or VSV-NRP ΔN; (C) wild-type Tax1 and either wild-type VSV-NRP or VSV-NRP D474N; (D) wild-type VSV-NRP and either wild-type Tax1 (wt) or mutant Tax1 in which the indicated lysine residues were mutated into arginine (K7–8R and K1–10R). Lysates were immunoprecipitated with an anti-VSV or anti Tax-1 antibody as indicated and western blot analyses were performed using anti-Tax1 and anti-VSV antibodies.

In order to map the domain(s) of NRP involved in the binding to Tax1, we performed co-immunoprecipitation experiments using a series of NRP mutants. We first co-expressed Tax1 with either a N-terminal or a C-terminal deletion mutant of NRP (Figure 3A). Immunoprecipitation of NRP ΔN alone led to the recovery of Tax1 from cell lysates (Figure 3B, upper panel, lane 3) while NRP ΔC was unable to interact with Tax1 (Figure 3B, upper panel, lane 2), showing that Tax1 binds to the C-terminal part of NRP. Immunoblotting for VSV-NRP confirmed that these mutants were expressed at similar levels when compared to the wild-type NRP (Figure 3B, lower panel), and both were efficiently immunoprecipitated with the anti-VSV antibody (Figure 3B, middle panel).

Because the C-terminal domain of NRP encompasses an ubiquitin-binding domain (UBD) (Figure 3A), we tested the contribution of this domain to the interaction with Tax1 (Figure 3C). Interestingly, a single point mutation in this domain known to disrupt the binding to K63-linked polyubiquitin (D474N) [25] was sufficient to severely reduce the interaction of NRP with Tax1 (Figure 3C, compare lane 1 and 2). This result suggests that NRP binding to Tax1 is mediated by an interaction between NRP UBD and K63-linked polyubiquitin chains conjugated to Tax1.

### Ubiquitination-defective Tax1 mutants exhibit impaired binding to NRP

Tax1 displays 10 lysine residues to which ubiquitination chains may be linked. It has been shown that lysines 4 to 8 significantly contribute to Tax1 polyubiquitination [15],[16],[26]. 293T cells were therefore transfected with Tax1 mutants in which all (K1–10R) or only a subset (K7–8R) of lysines were mutated into arginines. Interestingly, lysine-less Tax1 was impaired for NRP binding (Figure 3D, upper panel, lane 2), although this mutant was efficiently precipitated by the anti-Tax1 antibody (Figure 3D, middle panel, lane 2). Furthermore, the defect in NRP binding was not observed with mutant K7–8R (Figure 3D, upper panel, lane 3). These results strongly suggest that the integrity of Tax1 acceptor sites for ubiquitin is critical for the interaction with NRP. Taken together, these results further support the hypothesis that NRP binds to Tax1 through polyubiquitin chains on Tax1.

### Binding of NRP stabilizes Tax1 polyubiquitination

**Figure 4:**
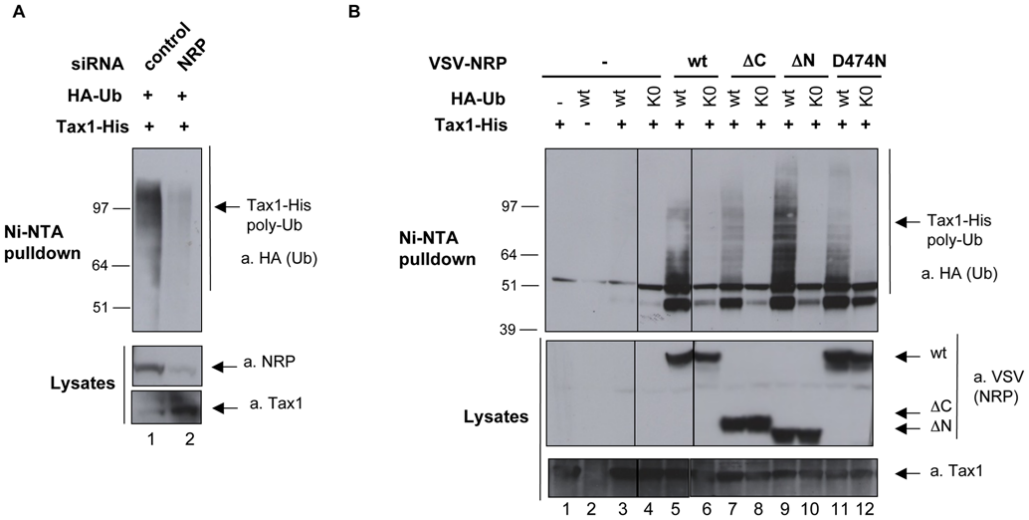
Modulation of Tax1 ubiquitination by NRP. (A) 293T cells were transfected with Tax1-His and HA-ubiquitin (HA-Ub), and either with an irrelevant siRNA directed against β-globin (control) or NRP-directed siRNA. (B) 293T cells were transfected with Tax1-His, and either wild-type HA-Ub or lysine-less HA-Ub (HA-Ub K0), together with wild-type VSV-NRP, VSV-NRP ΔC, VSV-NRP ΔN or VSV-NRP D474N, as indicated. Ubiquitinated forms of Tax1 were retained on Ni-NTA beads and processed for western blot analysis using anti-HA antibodies. Levels of expression of Tax1 and NRP in total lysates were determined by western blot using anti-Tax1 and (A) anti-NRP or (B) anti-VSV antibodies.

Tax1 ubiquitination is critical for its binding to the NEMO/IKK complex and its subsequent activation [15],[16],[18]. Given that NRP binds to polyubiquitinated Tax1, we wondered whether it could modulate Tax1 polyubiquitination status, and hence its ability to activate the NEMO/IKK complex. We thus analyzed the effect of silencing NRP on the level of polyubiquitinated Tax1. His-tagged Tax1 and HA-tagged ubiquitin were co-expressed in 293T cells, with a control siRNA (Figure 4A, lane 1) or with a siRNA targeting NRP (Figure 4A, lane 2). Ni-NTA pulldown was then performed in highly reducing and denaturating conditions, in order to avoid any deubiquitination and to ensure that only products covalently linked to Tax1 would be purified. By blotting for HA-ubiquitin, we assessed the level of polyubiquitinated Tax1 in each sample, which appears as high-molecular-weight products. When compared to control cells, the level of polyubiquitinated Tax1 was strikingly reduced in NRP-silenced cells, (Figure 4A, upper panel, compare lane 1 and 2). As control, we analyzed the level of NRP in cell lysates, and showed that it was indeed reduced in cells transfected with NRP-directed siRNA (Figure 4A, lower panel, compare lane 1 and 2). Thus, in the absence of NRP, polyubiquitinated Tax1 is less abundant.

One model that could account for this observation is that polyubiquitinated Tax1 is stabilized through its interaction with NRP. To test whether the effect of NRP on Tax1 polyubiquitination was dependent upon the interaction between both proteins, we performed the same type of experiments in cells over-expressing wild-type or mutant forms of NRP (NRP ΔN, ΔC and D474N) (Figure 4B). We predicted that wild-type NRP or NRP ΔN, which are able to bind to Tax1, would stabilize polyubiquitinated Tax1, whereas NRP ΔC and NRP D474N, which have lost the potential to bind to Tax1, would not. In order to highlight the differences among the lanes, a very short exposure time is shown, which accounts for the weak HA signal obtained in the absence of over-expressed NRP (Figure 4B, upper panel, lane 3). However, longer exposures revealed the presence of polyubiquitinated Tax1 in this lane (data not shown). As expected, we observed a correlation between the ability of NRP variants to bind to Tax1, and the level of polyubiquitinated Tax1 (Figure 4B, upper panel, compare lanes 5 and 9 with 7 and 11). However, we also observed that the levels of polyubiquitinated Tax1 were enhanced in cells over-expressing NRP ΔC or NRP D474N when compared to cells expressing endogenous NRP only (Figure 4B, compare lanes 7 and 11 to lane 3). This might be the consequence of a potential residual interaction between these mutants and Tax1.

To determine the linkage specificity of Tax1 polyubiquitination, we used an ubiquitin mutant (HA-Ub K0), in which all lysine residues are mutated to arginine (HA-Ub K0) and that can therefore no longer build conventional polyubiquitin chains. As expected, the HA-Ub K0 did not support Tax1 ubiquitination, indicating that the high-molecular-weight products of Tax1 indeed represented polyubiquitin chains (Figure 4B, lanes 4, 6, 8, 10 and 12). Altogether, these data suggest that NRP binds and stabilizes the polyubiquitin chains linked to Tax1.

### NRP potentiates Tax1-induced NF-κB activation

**Figure 5:**
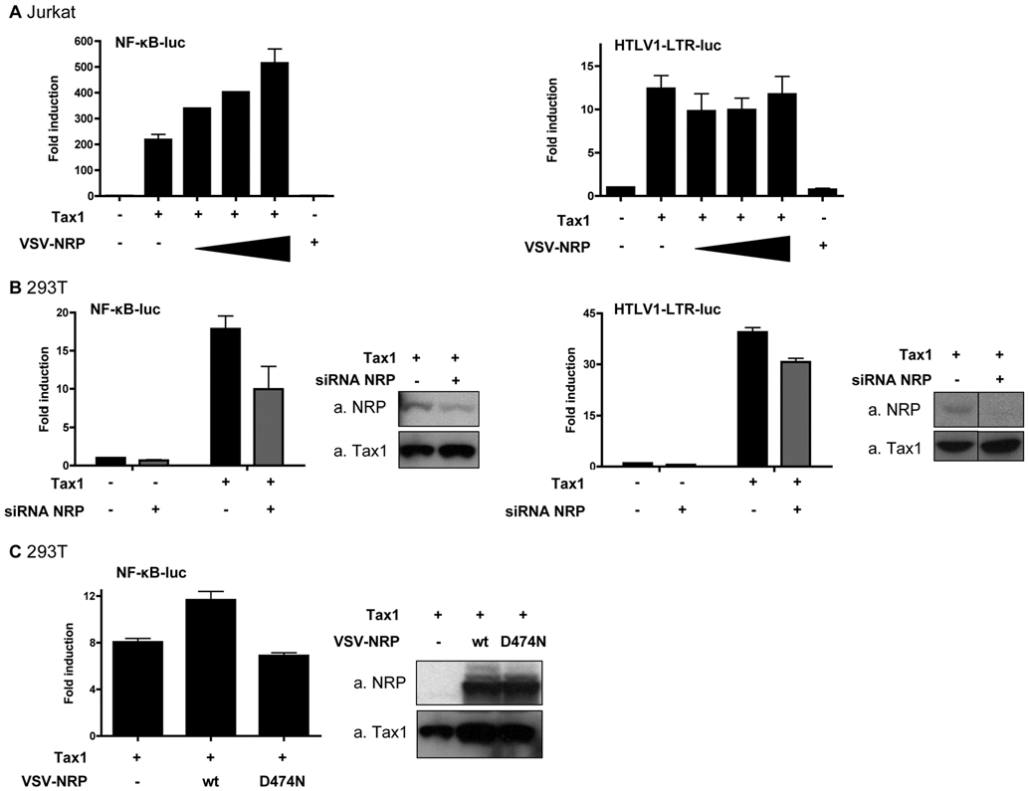
Potentiation of Tax1-dependent NF-κB activation by over-expressed and endogeneous NRP. (A) Jurkat T cells were transfected with either an Igκ-(κB)_3_-luc construct (left), or an HTLV-1-LTR-luc construct (right), together with Tax1 (1 µg) and VSV-NRP (range 0.25 to 1 µg) as indicated. (B) 293T cells were transfected with either an Igκ-(κB)_3_-luc construct (left), or an HTLV-1-LTR-luc construct (right), together with Tax1 (100 ng) and an irrelevant siRNA directed against β-globin (−) or NRP-targeted siRNA (+), as indicated. (C) 293T cells were transfected with an Igκ-(κB)_3_-luc construct together with Tax1 (400 ng) and either wild-type VSV-NRP or VSV-NRP D474N (400 ng) as indicated. (A, B, C) Luciferase activity was measured and normalized, and is shown as fold induction compared to basal promoter activity. (B, C) Cell lysates were run and probed for NRP and Tax1.

Because previous studies have suggested that Tax1 ubiquitination is critical for its ability to activate the NF-κB pathway [15],[16], we hypothesized that stabilization of Tax1 polyubiquitination by NRP would enhance activation of this pathway. We therefore tested the effect of increasing or decreasing NRP expression on Tax1-induced NF-κB activation using an NF-κB reporter gene assay (Figure 5). Jurkat cells were transfected with an NF-κB reporter gene together with Tax1 and increasing amounts of VSV-tagged NRP. As previously reported [5], NF-κB activity was induced upon Tax1 over-expression, and VSV-NRP further enhanced NF-κB activity in a dose-dependent manner (Figure 5A, left panel). We then evaluated the effect of NRP knockdown on Tax1-induced NF-κB activity. Compared with control siRNA, a two-fold decrease in Tax1-mediated NF-κB activity was observed when expression of NRP was silenced (Figure 5B, left panel), whereas no significant impact on the basal activity of the promoter could be measured in the absence of Tax1.

To ensure the specificity of NRP's effect on Tax1-mediated NF-κB activation, we performed the same experiments using a HTLV-1-LTR-luc reporter plasmid, which is known to be under the control of CREB rather than NF-κB. Using this construct, no or only a limited effect of NRP over-expression or silencing was observed (Figure 5A, right panel, and Figure 5B, right panel). As a control, NRP expression was determined in the presence or absence of siRNA directed against NRP (Figure 5B, left and right panels). Thus, NRP specifically enhances the activity of Tax1 on the NF-κB pathway.

We also tested whether the potentiating effect of NRP on Tax1-dependent NF-κB activity was dependent upon the interaction between Tax1 and NRP. Because we showed that NRP-D474N was impaired for the binding to Tax1 (Figure 3C) as well as for the stabilization of Tax1 polyubiquitination (Figure 4B), we compared the ability of wild-type NRP and NRP-D474N to potentiate Tax1 activity on the NF-κB reporter plasmid. As expected, NRP-D474N had no effect on Tax1 activity when compared to wild-type NRP, although expression levels of both constructs were similar (Figure 5C). Taken together, these results indicate that NRP specifically modulates Tax1-induced NF-κB activation, possibly by stabilizing Tax1 polyubiquitination in an interaction-dependent manner.

### TAX1BP1 cooperates with NRP for binding to Tax1 and modulation of Tax1 ubiquitination

Since we observed that NRP stabilizes Tax1 poly-ubiquitination, we wondered whether Tax1-binding protein 1 (TAX1BP1), which is also involved in ubiquitin-dependent regulation of NF-κB, could participate in this process. TAX1BP1 was originally identified as a binding partner of Tax1 [19],[20]. More recently, TAX1BP1 was reported to interact with A20, Itch and RNF11 to form a functional ubiquitin-editing complex that regulates the ubiquitination of RIP1 and TRAF6 [27]. Thus, we hypothesized that TAX1BP1 acts together with NRP to modulate Tax1 ubiquitination.

**Figure 6:**
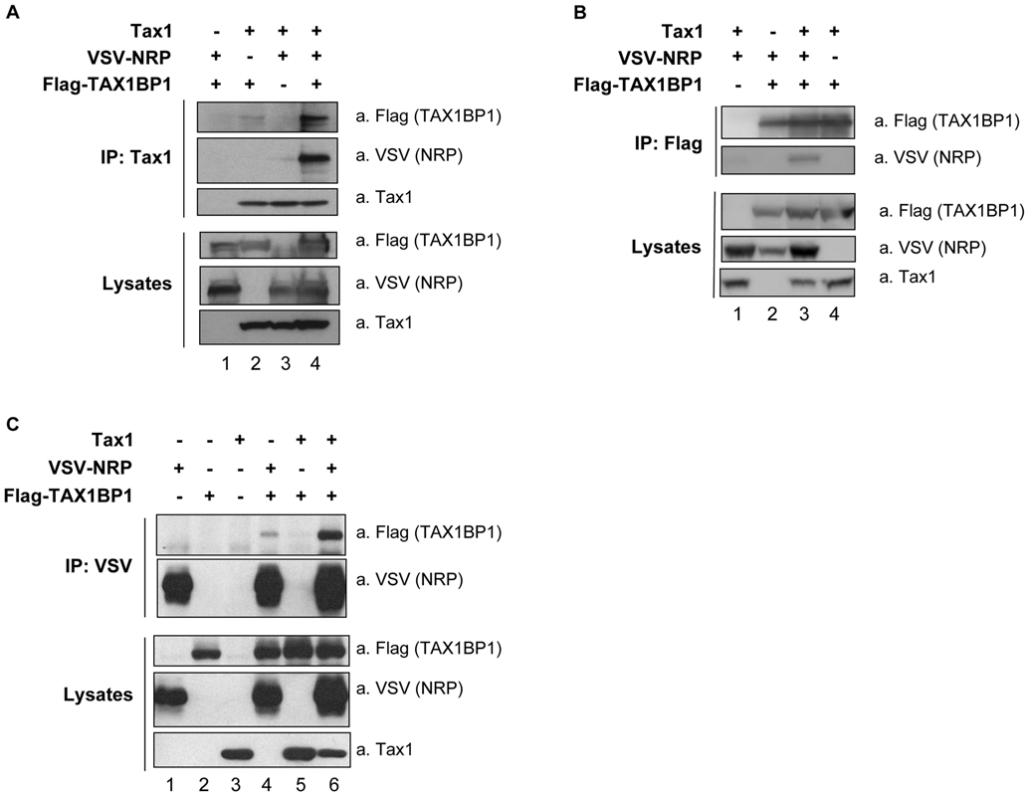
Synergistic interaction between Tax1, TAX1BP1 and NRP. 293T cells were transfected with Tax1, VSV-NRP and Flag-TAX1BP1 as indicated. Total lysates were immunoprecipitated with (A) anti-Tax1, (B) anti-Flag or (C) anti-VSV antibodies. Immunoblot analyses of immunoprecipitates and lysates were performed using antibodies directed against Flag, VSV and Tax1, as indicated.

293T cells were cotransfected with VSV-tagged NRP, Flag-tagged TAX1BP1 and Tax1, and immunoprecipitation of either Tax1, Flag-TAX1BP1 or VSV-NRP was performed (Figure 6A, B and C, respectively). Immunoprecipitates were then blotted with anti-Flag, anti-VSV or anti-Tax1 antibodies. These experiments confirmed that TAX1BP1 interacts with Tax1 (Figure 6A, lane 2). In addition, we observed that TAX1BP1 also interacts with NRP (Figure 6C, lane 4). More interestingly, the amount of TAX1BP1 associated with Tax1 was increased when NRP was co-expressed (Figure 6A, compare lanes 2 and 4). Similarly, the interaction between NRP and Tax1 was strongly induced in the presence of TAX1BP1 (Figure 6A, compare lanes 3 and 4) and the interaction between NRP and TAX1BP1 was also increased by the expression of Tax1 (Figure 6B, compare lanes 2 and 3, and Figure 6C, compare lanes 4 and 6). Thus, these results demonstrate that these three proteins interact with each other and suggest that NRP can be part of a ternary complex with Tax1 and TAX1BP1.

To study the functionality of this complex, we used several approaches. First, we performed immunofluorescence imaging to determine whether NRP or TAX1BP1 alone or in association could affect the localization of Tax1 to the Golgi apparatus and the recruitment of the NEMO/IKK complex by Tax1 to this organelle. Because NRP is localized at the Golgi apparatus, we first asked whether Tax1 localization to the Golgi-associated structures was dependent upon NRP expression. NRP-specific siRNA was used to specifically knockdown NRP expression in HeLa cells and Tax1-GFP localization was then investigated (Figure S3A and S3C). Silencing NRP did not impair the localization of Tax1-GFP to the Golgi apparatus (compare Figure S3B and S3C, upper panel), where it was still able to colocalize with NEMO (Figure S3C, lower panel). We then tested the effect of depleting either TAX1BP1 alone (Figure S3A and S3D) or together with NRP (Figure S3A and S3E) on the localization of Tax1 and NEMO to the Golgi apparatus. Preventing the expression of TAX1BP1 or of both TAX1BP1 and NRP had no effect on the subcellular distribution of Tax1 and NEMO (compare Figure S3B, S3D and S3E). Collectively, these results suggest that NRP and TAX1BP1 are not critical for the localization of Tax1 and for the recruitment of NEMO/IKK complex to the Golgi apparatus.

**Figure 7:**
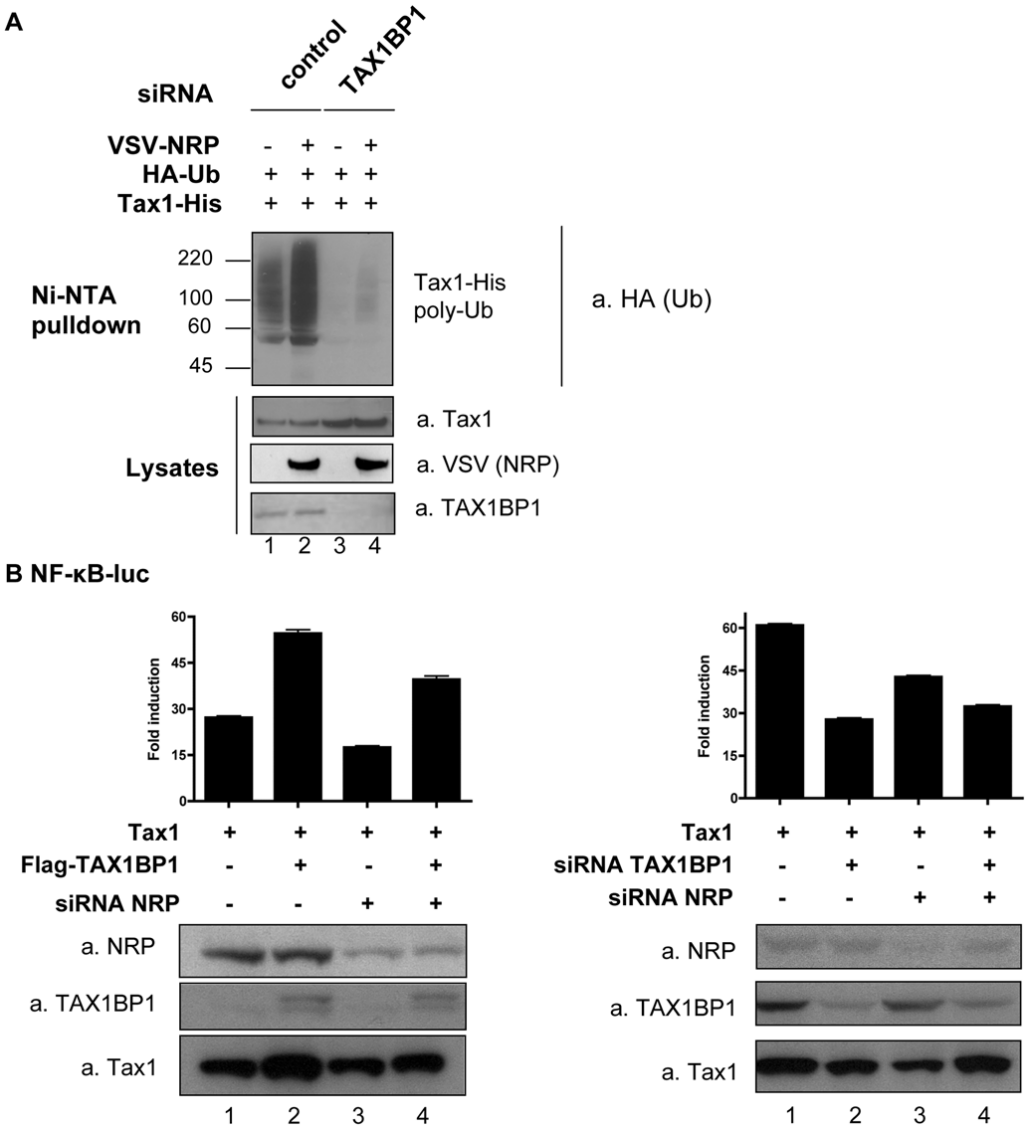
Cooperative modulation of Tax1 ubiquitination and NF-κB activation by NRP and TAX1BP1. (A) 293T cells were transfected with Tax1-His, HA-Ub, and VSV-NRP and either β-globin- (control) or TAX1BP1-directed siRNA, as indicated. Ubiquitinated forms of Tax1 were retained on Ni-NTA beads and processed for western blot analysis using anti-HA antibodies. Levels of expression of Tax1, VSV-NRP and TAX1BP1 in total lysates were verified by western blot using anti-Tax1, anti-VSV and anti-TAX1BP1 antibodies, respectively. (B) 293T cells were transfected with an Igκ-(κB)_3_-luc construct and Tax1, together with (left panel): Flag-TAX1BP1 and irrelevant β-globin (−) or NRP-targeted (+) siRNA; or (right panel): β-globin, NRP or TAX1BP1-specific siRNA, or both siRNA, as indicated. Luciferase activity was measured and normalized, and is shown as fold induction compared to basal activity. Levels of NRP, TAX1BP1 and Tax1 were assessed in cell lysates.

In another approach, we determined whether depleting TAX1BP1 could affect the regulatory effect of NRP on Tax1 ubiquitination and NF-κB activation (Figure 7). Interestingly, silencing TAX1BP1 expression precluded the stabilization of Tax1 ubiquitination that was observed when over-expressing NRP (Figure 7A, compare lanes 2 and 4). In addition, over-expressed TAX1BP1 up-regulated Tax1-dependent NF-κB activation (Figure 7B, left panel, lane 2) and this effect was decreased by silencing NRP (Figure 7B, left panel, lane 4). Furthermore, to determine whether NRP and TAX1BP1 act synergistically or not, we examined the effect of double-siRNA knock-down of NRP and TAX1BP1 on the activation of NF-κB mediated by Tax1. As expected from the ubiquitination assay (Figure 7A), we observed that depletion of TAX1BP1 decreased Tax1-mediated NF-κB activation (Figure 7B, right panel, lane 2). Interestingly, following the double depletion, the level of NF-κB activation was not further decreased as compared to the single depletion of TAX1BP1 (Figure 7B, right panel, lane 4). As controls, NRP, TAX1BP1 and Tax1 expression levels were determined by western blot (Figure 7A and 7B). Altogether, these results suggest that NRP and TAX1BP1 cooperate in Tax1-mediated NF-κB activation.

## Discussion

It is well established that one of the primary actions of Tax1 is to permanently activate the NF-κB signaling in the cytoplasm, and several models have been suggested to explain how this occurs. An important advance came first from experiments showing that Tax1 directly interacts with NEMO, which then functionally recruits Tax1 into the large NEMO/IKKα/IKKβ complex that phosphorylates IκB molecules [11]–[13]. How this interaction is regulated is not completely understood. To gain insight into this, we searched for additional Tax-interacting proteins. This led to the identification of NRP, also named Optineurin, as an interacting protein for Tax. This interaction is mediated through the ubiquitin-binding domain of NRP. Interestingly, mutations of the ubiquitination sites of Tax1 prevented its association with NRP, strongly suggesting that Tax1 ubiquitination is required for its interaction with NRP. Ubiquitination of Tax1 provides an important regulatory mechanism that promotes Tax1-mediated activation of NF-κB [15]–[17],[23]. Tax1 polyubiquitin chains are composed predominantly of K63-linked chains and the ubiquitination of Tax1 is dependent on the E2 ubiquitin-conjugating enzyme Ubc13 [17],[18]. The NF-κB activation process occurs through the direct binding of the ubiquitinated form of Tax1 to NEMO [16] in a specific subcellular compartment [18],[23],[28]. Different studies have suggested that Tax ubiquitination regulates IKK relocalization to the Golgi apparatus [23] or to the centrosome [18]. This relocalization involves the accumulation of Tax1 in Golgi-associated lipid rafts allowing the recruitment of the NEMO/IKK complex to these microdomains [28].

The interaction of NEMO with polyubiquitinated substrates is involved in transporting the NEMO/IKK complex towards its site of activation. This has been well described for the stimulation of NF-κB by the TNF-R and TCR [6],[29], allowing the recruitment of NEMO to these receptors and the subsequent phosphorylation of IKKβ by the upstream kinase TAK1. Similarly, DNA damage causes the translocation of NEMO from the cytoplasm to the nucleus and its phosphorylation by ATM [30]. Although previous studies have suggested that Tax1 ubiquitination is correlated with the localization of the NEMO/IKK complex at the Golgi apparatus, modulation of this process is still poorly understood. Our model is that NRP is a positive player in Tax1-induced NF-κB activation by increasing the polyubiquitination of Tax1. This model is supported by our results showing that increasing the level of NRP increases Tax1 polyubiquitination (Figure 4) and that the level of NRP expression correlates with Tax1-induced NF-κB activation (Figure 5). Whether NEMO also exerts a similar effect on Tax1 ubiquitination still remains unknown. Of note, a different effect of NRP was obtained with TNF-α-induced NF-κB activation, where NRP was shown to compete with NEMO for the binding to polyubiquitinated RIP and consequently to inhibit NF-κB activation [25]. The situation with Tax1 is unusual since the polyubiquitinated substrate that binds to NEMO and NRP is Tax1 itself, an NF-κB activator.

Concerning the regulation of Tax1 ubiquitination by NRP, the simplest interpretation of our experiments is that NRP either interacts directly or indirectly with an ubiquitin ligase, or that it prevents the interaction of Tax1 with a deubiquitinase. The E3 ubiquitin ligase(s) responsible for K63-linked polyubiquitination and the deubiquitinase responsible for the cleavage of these ubiquitin chains on Tax1 remain to be identified.

TAX1BP1 is a cellular protein that binds to Tax1 and acts as an ubiquitin-dependent negative regulator of NF-κB signaling in response to TNF-α stimulation [27]. It has recently been shown that this negative regulation is mediated by a quaternary complex containing TAX1BP1, A20, Itch and RNF11 [31]–[34]. Our results indicate that TAX1BP1 interacts with NRP and Tax1 individually and also with both proteins together to form a ternary complex, raising the possibility that TAX1BP1 participates in NRP-mediated enhancement of Tax1 ubiquitination. We have observed that the stabilization of Tax1 ubiquitination by NRP was completely impaired in the absence of TAX1BP1 (Figure 7A) and that NRP and TAX1BP1 cooperated to positively regulate Tax1-induced NF-κB activation (Figure 7B). Thus, we can propose that the negative regulation of TNF-α-induced NF-κB activation is mediated by a quaternary A20/TAX1BP1/Itch/RNF11 complex, as opposed to the positive regulation of Tax1-induced NF-κB activation, which is mediated by a ternary complex containing Tax1, TAX1BP1 and NRP.

Since it has been shown that Tax1 inactivates A20 by disrupting the TAX1BP1/Itch/A20 complex, thus counteracting its negative function [33], we speculate that NRP also is involved in this process. Furthermore, since RNF11 has been shown to interact with NRP [35], it will be important to determine whether this protein is also present in the TAX1BP1/Tax1/NRP complex and whether it regulates Tax1 ubiquitination. Future studies are needed to specifically address the mechanism whereby the Tax1/NRP/TAX1BP1 complex positively regulates Tax1 ubiquitination and subsequent NF-κB activation.

## Materials and Methods

### Cell culture

HeLa and 293T cell lines were grown in DMEM medium. HTLV-1-infected C8166 and C91PL and uninfected CEM and Jurkat cell lines were grown in RPMI 1640 medium. In all cases, the medium was supplemented with fetal bovine serum (10%) and antibiotics (100 units/ml penicillin and 100 µg/ml streptomycin), and cells were maintained at 37°C in 5% CO_2_.

### Constructs and siRNA

pSG5M-Tax1, pSG5M-Tax2, Tax1-6His, Tax1-GFP plasmids were previously described [18],[36]. Tax mutants harboring substitutions of all (K1–10R) or some (K7–8R) lysines into arginines were described elsewhere and were kindly provided by C. Pique [26]. HA- and VSV-tagged NRP plasmids were obtained by cloning NRP ORF (aa 1–577) into pT7link-HA or pcDNA3/pT7-link-GVSV vectors at EcoRI sites. To identify the domains of NRP required for *in vivo* interaction with Tax1, modified forms of NRP (VSV-NRP-ΔC (aa 1–278) and VSV-NRP-ΔN (aa 300–577)) (Figure 3A) were generated by site-directed mutagenesis with a PCR-based strategy and inserted at EcoRI sites into pcDNA3/pT7-link-GVSV vector. VSV-NRP D474N plasmid was generated by site-directed mutagenesis. HA-tagged wild-type and lysine-less (K0, in which all lysine residues are mutated into arginine) ubiquitin constructs were obtained from P. Jalinot [37]. Flag-tagged TAX1BP1 plasmid was a kind gift from E. Harhaj [33]. NRP double-stranded siRNA (GGAGACUGUUGGAAGCGAAGU) and β-globin double-stranded siRNA (control, GGUGAAUGUGGAAGAAGUU) were purchased from Proligo (Sigma). SMART pool siRNA directed against TAX1BP1 was purchased from Dharmacon.

### Antibodies

The following antibodies were used: anti-Tax1 (Tab172), anti-Tax2 (GP3738) [36], anti-NRP (100 000, Cayman Chemical), anti-HA (MMS-101R, Covance), anti-VSV (V 5507, Sigma-Aldrich, or ascite fluid of clone P5D4), anti-GM130 (610823, BD Transduction Laboratories), anti-NEMO (611306, BD Transduction Laboratories), anti-Flag M2 (F-3165, Sigma-Aldrich), anti-TAX1BP1 (sc-81390, Santa Cruz Biotechnology), anti-His (sc-804, Santa Cruz Biotechnology).

### Yeast two-hybrid screen

Yeast culture mediums were prepared and screens were performed as previously described [38]. Tax1 and Tax2 coding sequences were cloned by *in vitro* recombination using the Gateway® technology (Invitrogen) into Gal4-BD yeast two-hybrid vector pDEST32 (Invitrogen), and transformed into AH109 yeast strain (Clontech) using a standard Lithium/Acetate procedure. GAL4-BD-Tax1 and -Tax2 fusion proteins did not induce autonomous transactivation of *HIS3* reporter gene, and screens were performed on synthetic medium lacking histidine (−His medium) and supplemented with 10 mM of 3-amino-1,2,4-triazole (3-AT, Sigma-Aldrich). A mating strategy was used for screening the human spleen cDNA library cloned in the GAL4-AD pPC86 vector (Invitrogen) and previously transformed into Y187 yeast strain (Clontech). After 6 days of culture on selective medium, [His+] colonies were selected and purified over 3 weeks by culture on selective medium to eliminate false-positives. AD-cDNAs were amplified by PCR from zymolase-treated yeast colonies using primers that hybridize within the pPC86 regions flanking cDNA inserts. PCR products were sequenced and cellular interactors were identified by BLAST analysis.

### Transient transfections

293T cells were transfected using either the Polyfect reagent (Qiagen) or Lipofectamine 2000 (Invitrogen). For luciferase assays, 293T were first transfected with siRNA using Icafectin 442 (Eurogentec), followed 48 h later by DNA and siRNA transfection using Lipofectamine 2000. Jurkat cells were transfected using the Superfect reagent (Qiagen), except in Figure S2 where they were nuleofected using the Amaxa Nucleofector Technology (Amaxa Biosystems). HeLa cells were transfected using the Effectene reagent (Qiagen).

### Immunoprecipitations and immunoblots

293T, CEM, C8166 and C91PL cell lines cells were lysed in Chris buffer (50 mM Tris, pH 8.0, 0.5% Nonidet P-40, 200 mM NaCl, and 0.1 mM EDTA) supplemented with a cocktail of protease inhibitors (Complete, Roche), and the phosphatase inhibitors sodium fluoride (100 mM) and sodium orthovanadate (2 mM). Proteins were then recovered by immunoprecipitation from an equivalent amount of proteins, using one of the following antibodies: anti-Tax, anti-VSV, anti-NRP, anti-Flag. Immune complexes were recovered with magnetic Staphylococcus aureus Protein A or Protein G beads (Bio-Adembeads, Ademtech). Immunoprecipitates were then washed with lysis buffer, eluted and resolved by sodium dodecyl sulfate-polyacrylamide gel electrophoresis.

Subsequent immunoblots were performed according to a previously described protocol [36] and proteins transferred to nitrocellulose (I-Blot, Invitrogen) or Immobilon membranes (Millipore) were revealed with ECL Westen Blotting Substrate (Pierce) or ECL Plus Western Blotting Detection Reagent (Amersham).

### Glycerol gradient fractionation and gel filtration analysis

HeLa cells were lysed in a lysis buffer (50 mM Tris-HCl pH 7.4, 120 mM NaCl, 5 mM EDTA, 0.5% Nonidet-P40, 0.2 mM Na_3_VO_4_, 1 mM DTT, 1 mM PMSF) in the presence of protease inhibitors (Complete, Boehringer) by 15 passages through a 24-gauge needle.

Whole cell extracts were fractionated by centrifugation at 39000 rpm for 24 hrs on a 10–40% glycerol gradient (12 ml), using an Sw 41 rotor (Beckmann). The glycerol-containing buffers were prepared with the same composition as the lysis buffer. Twenty-four fractions (0.5 ml each) were then collected and 20 µl of each fraction were processed for immunoblot analysis.

For the gel filtration analysis, whole cell extracts were subjected to another centrifugation at 15000 rpm for 15 min, and then purified using Quick Spin Sephadex G25 columns (Roche). Gel filtration chromatography was carried out on a Superpose 6 column (Pharmacia) in a FPLC running buffer (50 mM Tris-HCl pH 7.4, 120 mM NaCl, 5 mM EDTA, 0.1% Nonidet-P40, 0.2 mM Na_3_VO_4_, 1 mM DTT, 1 mM PMSF, 10% glycerol) in the presence of protease inhibitors (Complete, Boehringer). The columns were precalibrated with albumin (67 kDa), aldolase (158 kDa), catalase (232 kDa), ferritin (440 kDa), thyroglobulin (669 kDa) and blue dextran (2000 kDa). Several glycerol gradients were loaded and analyzed with the same proteins, with the addition of the ovalbumin (43 kDa). Forty fractions of 0.5 ml each were then collected and 20 µl of fractions 16 to 40 were processed for immunoblot analysis.

### Nickel pull-down

Forty-eight hours after transfection, cells were harvested in cold PBS and lysed under highly denaturating and reductive conditions in Guanidium Buffer (10 mM Tris HCl pH 8.0, 100 mM Na_2_HPO_4_/NaH_2_PO_4_, 6 M Guanidium) [26]. Cell lysates were then incubated with Ni-NTA beads (His-select HF Agarose Beads, Sigma-Aldrich) at room temperature. Beads were then extensively washed with Guanidium Buffer, Urea Buffer (10 mM Tris HCl pH 6.4, 100 mM Na_2_HPO_4_/NaH_2_PO_4_, 8 M Urea) and cold PBS. Bound proteins were finally eluted and processed for immunoblot analysis.

### Indirect immunofluorescence

Twenty-four hours post-transfection, cells were fixed with 4% paraformaldehyde, rinsed and permeabilized in PBS containing 0.5% Triton X-100. Following pre-incubation with PBS containing 5% BSA, cells were incubated with primary antibodies in PBS containing 1% BSA for 1 h at room temperature. Samples were then stained with Alexa Fluor 568-conjugated goat anti-rabbit IgG (A-11010, Invitrogen), Cy5-conjugated donkey anti-mouse IgG (715-175-150, Jackson ImmunoResearch Laboratories), or AMCA-conjugated horse anti-mouse IgG (CI-2000, Vector Laboratories) for 1 h at room temperature. Where indicated, an additional staining of nuclei was performed with DAPI (Sigma) for 5 min. The coverslips were washed, mounted with Vectashield Mounting Medium (H-1000, Vector Laboratories), and examined under a Zeiss Axioplan 2 microscope, using the Zeiss ApoTome system and the Zeiss Axiovision 4.4 software.

### Luciferase assays

Jurkat and 293T cells were transiently transfected with either an HTLV-1-LTR-luc or an Igκ-(κB)_3_-luc plasmid together with the indicated plasmids or siRNA. The amount of total DNA was equalized using a pSG5M backbone vector, as previously reported [36]. All transfections were carried out in the presence of a renilla luciferase vector (phRG-TK) in order to normalize the results for transfection efficiency. Luciferase activity was assayed 18 h post-transfection using the Dual-Luciferase Reporter Assay System (Promega) on a Berthold LB9500C luminometer as reported previously [39].

## Supporting Information

Figure S1 Glycerol gradient analysis of Tax1 and NRP. HeLa cells were transfected with Tax1 and HA-NRP, and cell extracts were separated through a glycerol gradient. All fractions as well as cell extracts (input) were analyzed by western blotting using antibodies directed against HA (upper panel) or Tax1 (lower panel). Precalibration of the glycerol gradient is indicated beneath the fractions count (kDa).
(5.09 MB TIF)

Figure S2 Colocalization of Tax1, NRP and NEMO in Golgi-associated structures. Jurkat cells were transfected with (A and B) Tax1-GFP or (C) GFP alone and stained with an anti-NRP antibody (red) and either (A and C) an anti-GM130 or (B) an anti-NEMO antibody (blue). Cells were observed as described in the Materials and Methods section. Differential interference contrast (DIC) is shown. Scale bar = 10 µm.
(5.89 MB TIF)

Figure S3 Effect of NRP or TAX1BP1 silencing on Tax1 localization and colocalization with NEMO. HeLa cells were transfected with Tax1-GFP and with siRNA directed against either (A and B) β-globin (control, -), (A and C) NRP, (A and D) TAX1BP1, or (A and E) both NRP and TAX1BP1. (A) Lysates were analyzed by western blot to control NRP and/or TAX1BP1 depletion. (B to E) Cells were stained with either an anti-GM130 or an anti-NEMO antibody as indicated (red). Nuclei were stained using DAPI (blue). Cells were observed as described in the Materials and Methods section. Scale bar = 10 µm.
(9.35 MB TIF)
