# Supplementary figures and images for: NRP/Optineurin Cooperates with TAX1BP1 to Potentiate the Activation of NF-κB by Human T-Lymphotropic Virus Type 1 Tax Protein
Source: PLoS Pathog. 2009 Jul 17;5(7):e1000521. doi: 10.1371/journal.ppat.1000521 (PMC2706988; doi:10.1371/journal.ppat.1000521)

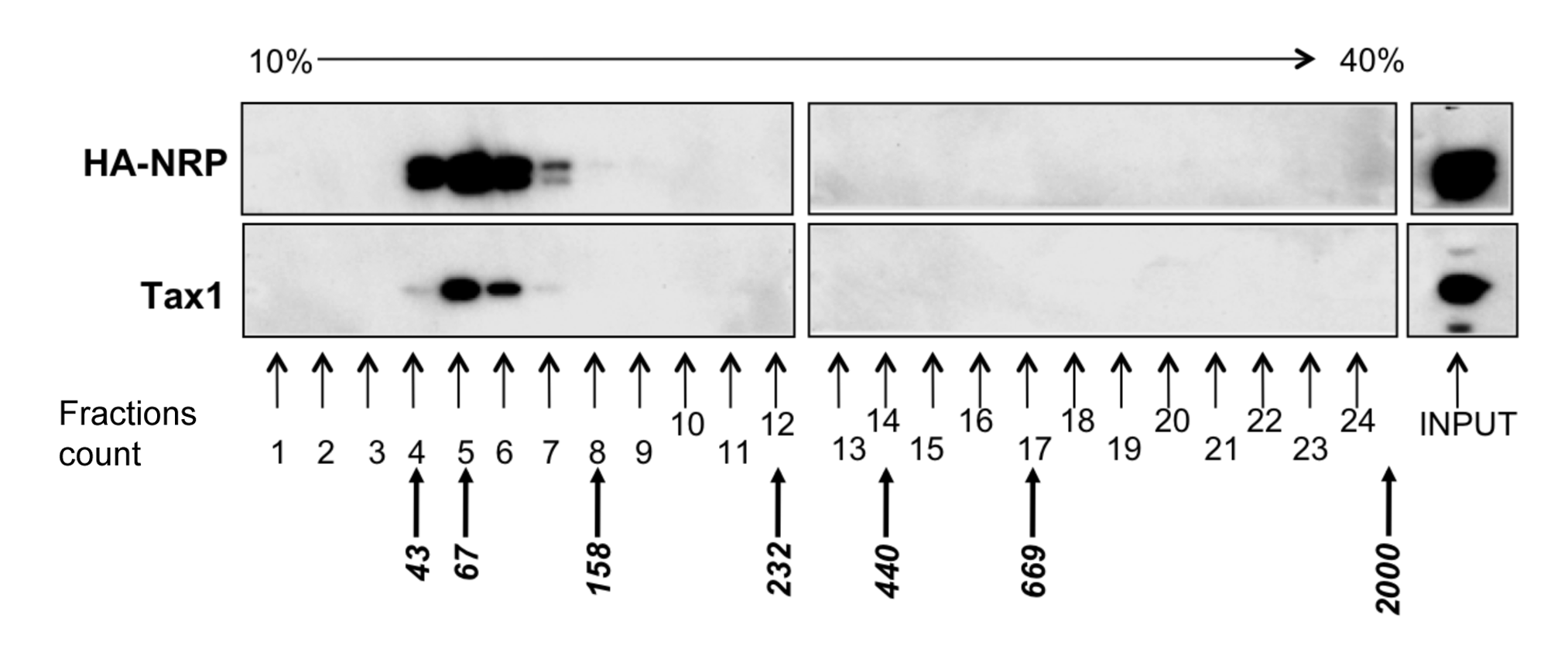

Supplement: Figure S1 — Glycerol gradient analysis of Tax1 and NRP. HeLa cells were transfected with Tax1 and HA-NRP, and cell extracts were separated through a glycerol gradient. All fractions as well as cell extracts (input) were analyzed by western blotting using antibodies directed against HA (upper panel) or Tax1 (lower panel). Precalibration of the glycerol gradient is indicated beneath the fractions count (kDa). (5.09 MB TIF) [file ppat.1000521.s001.tif]

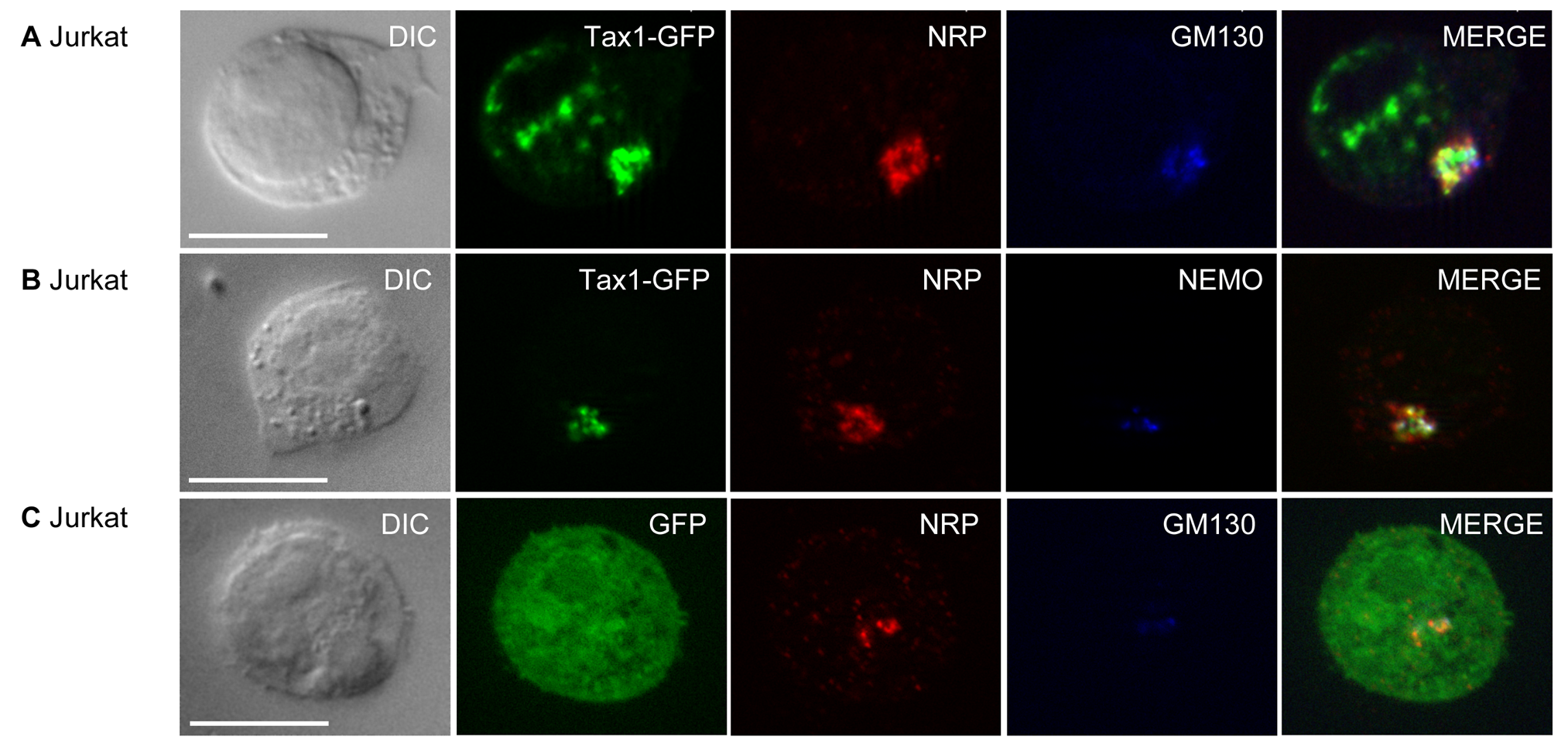

Supplement: Figure S2 — Colocalization of Tax1, NRP and NEMO in Golgi-associated structures. Jurkat cells were transfected with (A and B) Tax1-GFP or (C) GFP alone and stained with an anti-NRP antibody (red) and either (A and C) an anti-GM130 or (B) an anti-NEMO antibody (blue). Cells were observed as described in the Materials and Methods section. Differential interference contrast (DIC) is shown. Scale bar = 10 µm. (5.89 MB TIF) [file ppat.1000521.s002.tif]

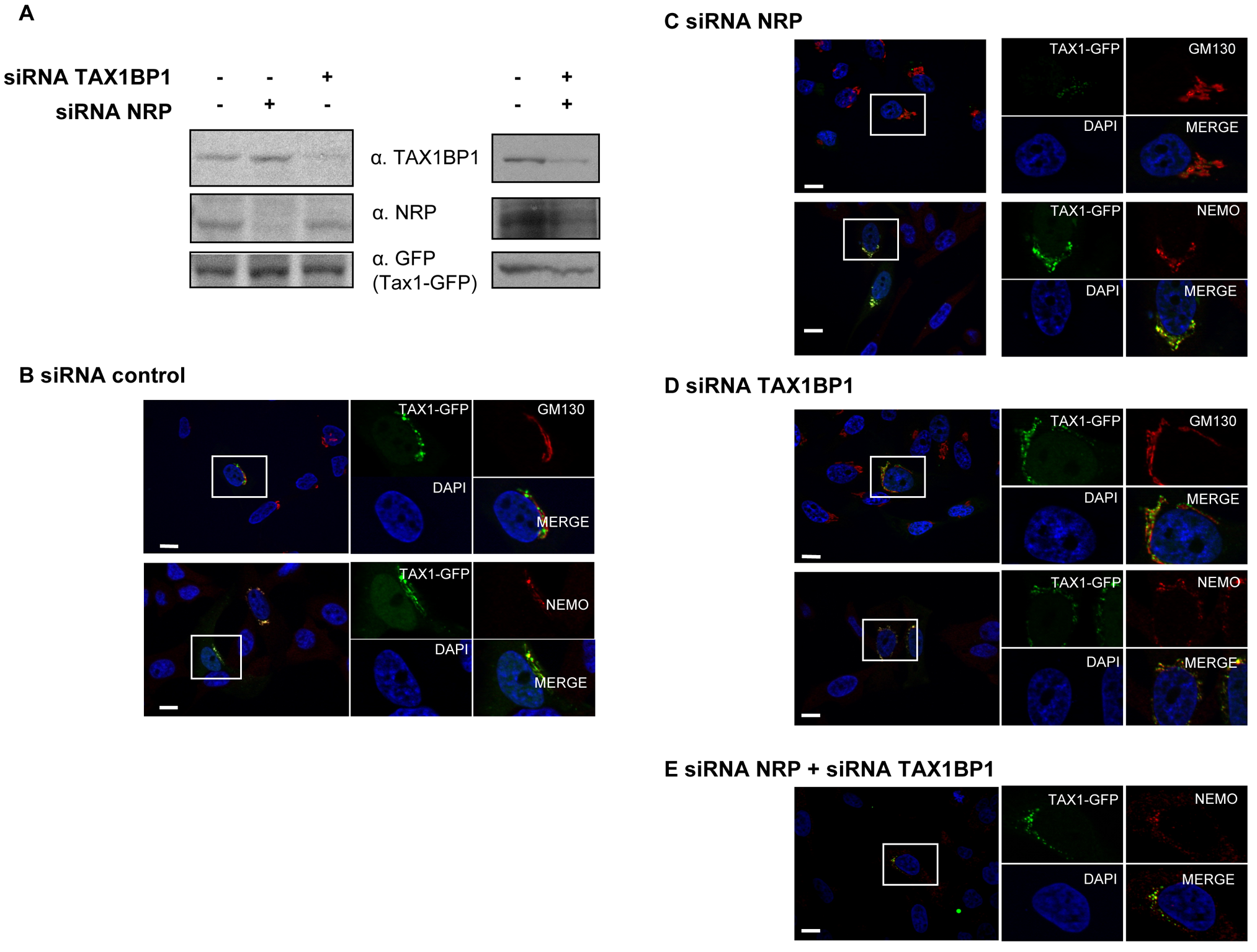

Supplement: Figure S3 — Effect of NRP or TAX1BP1 silencing on Tax1 localization and colocalization with NEMO. HeLa cells were transfected with Tax1-GFP and with siRNA directed against either (A and B) β-globin (control, -), (A and C) NRP, (A and D) TAX1BP1, or (A and E) both NRP and TAX1BP1. (A) Lysates were analyzed by western blot to control NRP and/or TAX1BP1 depletion. (B to E) Cells were stained with either an anti-GM130 or an anti-NEMO antibody as indicated (red). Nuclei were stained using DAPI (blue). Cells were observed as described in the Materials and Methods section. Scale bar = 10 µm. (9.35 MB TIF) [file ppat.1000521.s003.tif]
